# Effects of mindfulness decompression therapy on mental health and job burnout among nurses working in the frontline of the novel coronavirus pandemic: A retrospective study

**DOI:** 10.1002/1348-9585.12398

**Published:** 2023-04-10

**Authors:** Ling Xia Luo, Xiaobei Peng, Jianmei Hou, Yanhui Xie, Huiqian Dong, Sha Peng, Guiyuan Ma, Jinghui Zhang

**Affiliations:** ^1^ Teaching and Research Section of Clinical Nursing Xiangya Hospital of Central South University Changsha China; ^2^ Emergency Department Xiangya Hospital of Central South University Changsha China; ^3^ National Clinical and Medical Research Center for Geriatric Diseases (Xiangya Hospital) Xiangya Hospital of Central South University Changsha China; ^4^ Xiangya School of Nursing Central South University Changsha China

**Keywords:** front‐line nurses, job burnout, mental health, mindfulness decompression, nucleic acid sampling area

## Abstract

**Objectives:**

As the coronavirus disease 2019 (COVID‐19) pandemic continues to spread worldwide, nucleic acid detection is a key step in controlling it. Psychological issues and job burnout of nurses working in nucleic acid sampling roles for long periods have become apparent. This study aimed to explore the effects of mindfulness decompression therapy on mental health and job burnout in front‐line nurses working in nucleic acid sampling during the pandemic.

**Methods:**

Nucleic acid sampling frontline nurses who were positive for burnout on both the Symptom Checklist‐90 (SCL‐90) and the Maslach Burnout Inventory‐General Scale (MBI‐GS) were selected as the participants. Frontline nurses in the nucleic acid testing area who received routine psychological nursing intervention from June 2020 to April 2021 were used as the control group. Nurses who received both routine psychological nursing and mindfulness decompression therapy from May 2021 to December 2021 formed the “mindfulness” subject group. We compared the two groups' primary outcome measures of SCL‐90 and MBI‐GS scores.

**Results:**

Before the intervention, there were no significant differences between the two groups in general data, SCL‐90 scores, and MBI‐GS scores. After the mindfulness decompression therapy, according to SCL‐90 and MBI‐GS scales, psychological distress and job burnout of nurses in the mindfulness group were significantly better than those in the control group.

**Conclusion:**

Mindfulness decompression therapy can effectively improve mental health and relieve job burnout in frontline nurses in nucleic acid sampling areas, which is worthy of clinical application. Randomized controlled trials are still needed, however, to fully confirm the effects of mindfulness decompression therapy.

## 1 INTRODUCTION

Since January 30, 2020, the Coronavirus Disease 2019 (COVID‐19) has been a global public health emergency, as declared by the World Health Organization (WHO).^1^ According to the WHO's last update at the time of writing, July 2, 2022, at 00:25 am GMT + 8, there have been 545 226 550 confirmed cases of COVID‐19 globally, including 6 334 728 deaths and a case fatality rate of 1.16%.^2^ Nucleic acid testing is essential for the prevention and control of this deadly disease.^3^ In China, to facilitate normalized management and timely screening of virus carriers and confirmed COVID‐19 patients, all regions have strengthened nucleic acid testing, especially for citizens in medium and high‐risk areas.^4^ Nurses in the nucleic acid sampling area wear personal protective equipment (PPE), including clothing, masks, goggles, hoods, etc. throughout their workdays and may feel stuffy, airtight, or even dizzy while wearing them.^5^ The increased workload and physical burden of wearing PPE threaten nurses' mental health and increase their job burnout rates.^6^ Common psychological problems among nurses can manifest as intense somatization, anxiety, depression, obsessive symptoms, and fear.^7^

Mindfulness decompression therapy is a program that incorporates mindfulness to assist people with pain, as well as a range of conditions and life issues that are difficult to treat in a hospital setting. People use meditative practices to reduce psychological and physical stress;^8^ Mindfulness decompression therapy programs have been researched as potential interventions for reducing stress and burnout by cultivating present awareness, emotional regulation, and positive thinking.^9^ Previous research has shown that mindfulness programs have real effects on reducing burnout and mental health among healthcare workers. Studies on nurses have also demonstrated their positive impact.^10^ One study included a range of mindfulness‐based psychotherapies such as mindful eating, body scanning, mindful breathing, mindful meditation, mindful yoga, mindful walking, sitting meditation, and self‐exploration.^11^ mindfulness‐based psychotherapies are beneficial for correcting suboptimal health states, achieving emotional management, and relieving work pressure, and are effective for various groups of people.^12^ This study aimed to evaluate the effects of mindfulness‐based stress reduction interventions on improving the mental state and job burnout of frontline nurses in nucleic acid sampling areas.

## 2 METHODS

### 2.1 Study design

This retrospective study used the psychological monitoring data of front‐line clinical nurses in the nucleic acid detection area of our hospital's (Xiangya Hospital, Central South University, Changsha, China) psychological prevention and control team. Our hospital has established a psychological prevention and control implementation department composed of staff from the human resources department, nursing department, medical department, and mental health centre. The psychological intervention was provided, and the data were stored in a database. In this study, frontline nurses in the nucleic acid testing area who received routine psychological nursing interventions from June 2020 to April 2021 were included in the control group.

The nucleic acid testing nurses who received routine psychological nursing + mindfulness decompression therapy from May 2021 to December 2021, the first‐line nurses in the district, were labeled as the “mindfulness group”. From May to December 2021, every time we recruited 20–25 participants, we ran a mindfulness decompression therapy course. According to the study plan, participants were recruited into one group for no more than 1 month for each period. During this period, we recruited 65 volunteers to participate in the study [*n* = 65; age (years) range: (25–29): *n* = 29 (44.6%); (30–34): *n* = 22 (33.8%); male:12 (18.5%), female: 53 (81.5%)]. The same questionnaire was used for retesting after 8 weeks of mindfulness decompression therapy course intervention. The differences in the Symptom Checklist‐90(SCL‐90) and Maslach Burnout Inventory‐General Scale (MBI‐GS)scores of the nurses in the two groups before and after the intervention were compared. This study was reviewed and approved by the ethics committee of our hospital (approval number: 202005402).

### 2.2 Participants

Inclusion criteria: (1) Informed consent and voluntary participation in the study; (2) registered nurses (referred nurses who had obtained the “Nurse Practitioner Qualification Certificate” in China) over 20 years old; (3) working in front‐line work in nucleic acid sampling areas; (4) SCL‐90 scores (subscale score higher than 2, positive items higher than 43, or total score higher than 160) and MBI‐GS scores (>75 points) both positive. Exclusion criteria: (1) those who did not receive drug intervention; and (2) those who were lost to follow‐up. Elimination criteria: (1) Participants who did not adhere to the intervention steps within each group (i.e., participants who met the inclusion criteria for our study and were enrolled in the study, but by the end had incomplete data; participants who did not fully participate in mindfulness therapy for 8 weeks, etc.).

### 2.3 Questionnaire and assessment tools

The questionnaire consisted of three parts: basic demographic information, a job burnout assessment by the MBI‐GS, and a mental health assessment by the SCL‐90. Sociodemographic characteristics included sex, age, education, marital status, years of work, occupational qualifications, time spent working in a nucleic acid sampling unit, and level of concern of subject/subject's family being infected with COVID‐19.

Mental health was evaluated using the SCL‐90^13^, ^14^ scale, which comprises 90 items, each made up of 10 factors. Each item is scored on a 5‐point Likert scale ranging from 0 (none) to 4 (severe), with a total of 10 dimensions including anxiety, depression, paranoia, hostility, terror, interpersonal sensitivity, obsessive–compulsive symptoms, psychosis, somatization, and sleep disorders. The higher the total scores the nurses received, the worse their mental health condition. Each factor had good internal consistency reliability, and the coefficient of Cronbach's α was 0.92.^13^, ^14^ In this study, Participants participated in the survey on a self‐reported basis, a statistical analysis was performed on the total score and each latitude score of the SCL‐90. If any subscale score was higher than 2, positive items were higher than 43, or the total score was higher than 160, it suggested a psychological abnormality.

Job burnout was evaluated using the MBI‐GS,^15^ for which usage permission was obtained through the official channels. The MBI‐GS is a modified version of the original MBI specifically designed to assess burnout in any occupational setting. We decided to use the MBI‐GS, as this scale is based on the assumption that burnout takes the same form in all occupational sectors and is related to the general performance of work, rather than to relationships at work (e.g., with patients)—that is, burnout can occur anytime and anywhere there is a major imbalance, or mismatch, between demands in the work environment and the individual's available resources.^15^ MBI‐GS^16^ includes three dimensions: emotional exhaustion, work indifference, and common sense of achievement. The Likert 7‐level scoring method was used: “0” for “never” and “6” for “very often.” Emotional exhaustion (5 items) covers the experience of both emotional and physical fatigue; cynicism (5 items) reflects indifference, detached attitude towards work, and active disengagement from work; and professional efficacy (6 items) consists of feelings of competence, achievement, and accomplishment in one's work, which diminish when burnout is developing. The MBI‐GS measures emotional exhaustion, cynicism, and personal accomplishment, with higher scores on exhaustion and cynicism subscales indicating higher levels of burnout. In contrast, a lower score on personal accomplishment indicates burnout due to decreased motivation. The MBI‐GS is scored on a scale of 0–6 points from “no” to “always” for questions 1–8, and Questions 9–15 are scored on a scale of 6–0 from “no” to “always”. The sum of these items was the scale score. According to the score, the degree of burnout was divided into four levels: (1) Below 50 points: Good working conditions; (2) 50–75 points: There is a certain degree of job burnout and a need for psychological adjustment; (3) 75–100 points: It is recommended the subject take vacation and leave the workplace for some time to adjust; (4) More than 100 points: suggested that the subject stake counseling, quit, cease working, or change jobs for a more positive life. The adjusted MBI‐GS structure is consistent with the original, which shows that the MBI‐GS has good conceptual validity in China. The internal consistency coefficients for emotional exhaustion, cynicism, and reduced personal accomplishment were 0.88, 0.83, and 0.82 respectively.^15^, ^16^

#### 2.3.1 Routine nursing intervention

The pre‐job training for nurses in nucleic acid sampling area includes occupational safety protection training, novel coronavirus prevention and control guidelines (daily work content of COVID‐19 prevention and control), and nucleic acid sampling‐related emergency plan training (such as learning how to properly put on and take off personal protective equipment). Hospital nursing department, infection management Center, and network information Center to participate in this work. (1) Strengthen communication: guided nucleic acid sampling nurses to express themselves courageously, listen patiently to their difficulties, and helped them solve problems; (2) Emotion management: trained nucleic acid sampling nurses to relieve their stress through reading, exercise, diet adjustment, etc.^17^ On the premise of receiving the above training, front‐line nurses in the nucleic acid sampling area were included in the study and treated with mindfulness decompression therapy for 8 weeks.

#### 2.3.2 Mindfulness decompression therapy

This study established a mindfulness decompression therapy group, which included a mental health outpatient specialist with 5 years of clinical work experience, responsible for the formulation and quality control of intervention programs. Three psychological counselors with the national second‐level psychological counselor certificate, were responsible for implementing the intervention plan, and two postgraduate students were responsible for questionnaire surveys and follow‐up. To develop a mindfulness decompression therapy manual, mindfulness decompression therapy audio was recorded and distributed to each enrolled nurse. Psychological counselors guided the nurses in conducting mindfulness decompression therapy. Three mindfulness decompression therapy courses (the content of the three courses was the same and completed by a team of three counselors) were formulated and implemented through the mindfulness intervention group, which was held in the staff activity centre (conference room) of a hospital in Changsha City, Hunan Province, China. From May to December 2021, participants were enrolled in cohorts of 20–25, and eight‐week mindfulness decompression therapy courses were conducted by our trained team. The courses took place on Friday evenings at 6 p.m., for 2 h. Participants were also asked to practice at home for 15–30 min every day and log into the course's WeChat group. Participants enrolled in the routine psychological nursing intervention were excluded from the regular psychological nursing + mindfulness decompression therapy group. The study enrolled 65 participants; 25 completed the first course, 22 completed the second course, and 18 completed the third course. Before mindfulness decompression therapy, each participant in the mindfulness group received a copy of “Eight‐Week Mindfulness Journey” compiled by British psychologists John Teasdale and Mark Williams. The intervention plans are listed in Table 1. At home, participants practiced mindfulness decompression therapy courses for 5–15 min every day, according to the audio guide. Participants were free to extend these practice times according to their wishes and log into the mindfulness decompression therapy WeChat control group after each completion.

**TABLE 1:** Contents of mindfulness decompression therapy.

Time	Items	Main contents and methods	Homework
Week 1	Mindful Eating	Guide the nurses to eat raisins: make the nurses focus on the present moment, pay attention to the appearance, touch the texture, and smell the grapes, and promote the nurses to switch from the automatic navigation mode to the mindful mode	Completed mindfulness diet each day and recorded them in the mindfulness decompression treatment manual^a^ (Video S1)
Week 2	Body Scan	Body scanning exercises: the nurses lie on their backs, relax their bodies, close their eyes, focus on each part of the body in order, and scan the body from bottom to top, focusing on the physical sensations of the moment	Completed body scanning exercises at least 6 days a week and recorded them in the mindfulness decompression treatment manual
Week 3	Mindful Breathing	Guide the nurse to carry out mindful breathing exercises: close your eyes, do not deliberately control the breathing rate, quietly experience each breath with the instructor, and let those bad emotions dissolve in each airflow	Completed mindfulness breathing exercises at least 6 days a week and recorded them in the mindfulness decompression treatment manual
Week 4	Mindfulness Meditation	Guide the nurses to let go of their thoughts, put themselves in the blue sea, quiet forest, beautiful picture scroll, or wide desert, and pay attention to the self and physical experience at this time	Completed mindfulness meditation exercises at least 6 days a week and recorded them in the mindfulness decompression treatment manual (Video S2)
Week 5	Mindfulness Yoga	Practice mindfulness yoga: the purpose is to make the nurse aware of the physical and mental reality, achieve a state of physical and mental unity and harmony between man and nature, which can effectively make the nurses feel quiet and peaceful	Completed mindfulness yoga exercises at least 6 days a week and recorded them in the mindfulness decompression treatment manual
Week 6	Mindful Walking	Perform mindfulness walking: mindfulness walking is to conduct mindfulness training during walking. Its purpose is to enable nurses to experience this physical sensation and maintain awareness during walking	Completed mindfulness walking exercises at least 6 days a week and recorded them in the mindfulness decompression treatment manual
Week 7	Sitting Meditation	Practice mindfulness meditation: When the attention is taken away, recognize the thought at the moment, and then remind yourself that this is just a “thought”, gently bringing the attention back to the breath	Completed mindfulness meditation exercises for at least 6 days a week and recorded them in the mindfulness decompression treatment manual
Week 8	Self‐exploration	Review and summarize the 8 weeks of study and experience, share my feelings with the people around me, re‐understand myself, accept myself, and integrate mindfulness concepts into my future life	Mindfulness self‐exploration training, build your own mindfulness practice schedule based on your actual situation

^a^ The mindfulness decompression treatment manual—Specifically to document the experience of undergoing mindfulness decompression therapy.

During mindfulness decompression therapy, the psychiatrist and psychological counselor of the research team were responsible for comprehensively understanding the psychological state of each nucleic acid sampling nurse, strengthening communication with each participant, and answering the psychological problems of each participant during the intervention. Psychological counselors held regular discussions and exchange meetings and provided corresponding discussions and feedback on the steps of mindfulness decompression therapy. They also dynamically observed the mental state of each enrolled nurse, identified those with abnormal moods, increased their individual treatment plans, and referred them if necessary. The psychologist was responsible for quality monitoring and implementing mindfulness intervention, as well as discussing and adjusting tratment promptly when problems were found. Each group had full‐time staff responsible for contacting and informing the participants to participate in group counseling, ensuring that they participated in activities on time throughout the process, and recording cases of loss. After each mindfulness intervention, a satisfaction survey was performed, feedback was obtained, discussions were initiated, and adjustments were made. Research participants who encountered severe psychological barriers received timely one‐on‐one consultations or referrals.

### 2.4 Data analysis

The statistical analyses were performed using the Statistical Package for the Social Sciences (IBM SPSS Statistics for Windows, IBM Corp.) software, version 26.0. The general demographic data were described in terms of frequency, percentage, mean, and standard deviation (mean ± SD). Using the χ^2^ test, an independent sample Student's *t*‐test was used to compare differences between the groups. Measurement data conformed to a normal distribution, expressed as ($\bar{x}$±s), using an independent sample *t*‐test to compare differences between groups, and paired sample *t*‐test to compare scores within the group. *P* < .05 was taken to indicate that differences were statistically significant.

## 3 RESULTS

### 3.1 Demographic and job‐related characteristics

Table 2 provides the demographic and job‐related characteristics of the nurses who participated in this study. Of the 130 participants, 108 (83.07%) were female, and the proportions of participants aged 25–29 years and 30–34 years old were 43.85% and 31.54%, respectively. Of these, 68.46% of the nurses' professional titles were primary nurses. The most common (*n* = 105, 80.77%) educational qualification was undergraduate. Most had been employed for more than 60 days (*n* = 73, 56.15%). The Chi‐square test showed no significant differences in sex, age, education, working years, professional title, marital status, or nucleic acid sampling working time between the groups(all *P*‐values >.10).

**TABLE 2:** General demographic data and comparison between the two groups (*n* = 130).

	*n* (%) of participants		
Variable	Control group (*n* = 65)	Mindfulness group (*n* = 65)	*P*
Gender			
Male	10 (15.4%)	12 (18.5%)	.643
Female	55 (84.6%)	53 (81.5%)	
Age, years			
<25	11 (16.9%)	4 (6.1%)	.707
25–29	28 (43.0%)	29 (44.6%)	
30–34	19 (29.2%)	22 (33.8%)	
35–39	4 (6.1%)	7 (10.7%)	
≥40	3 (4.6%)	3 (4.6%)	
Marital status			
Never	27 (41.5%)	32 (49.2%)	.494
Married	37 (56.9%)	32 (49.2%)	
Divorced	1 (1.5%)	1 (1.5%)	
Educational level			
Junior college	9 (13.8%)	7 (10.7%)	.471
Undergraduate	52 (80.0%)	53 (81.5%)	
Master degree	4 (6.1%)	5 (7.6%)	
Professional qualification			
Primary	45 (69.2%)	44 (67.6%)	.236
Middle	19 (29.2%)	18 (27.6%)	
Senior	1 (1.5%)	3 (4.6%)	
Length of working experience			
<5	19 (29.2%)	20 (30.7%)	.131
5–9	24 (36.9%)	32 (49.2%)	
10–19	19 (29.2%)	11 (16.9%)	
>20	3 (4.6%)	2 (3.0)	
Number of days worked in nucleic acid sampling area			
<15	5 (7.6%)	6 (9.2%)	.106
15–30	6 (9.2%)	17 (26.1%)	
31–60	12 (18.4%)	11 (16.9%)	
>60	42 (64.6%)	31 (47.6%)	

### 3.2 Comparison of psychological status between the two groups of participants before and after intervention

Before the intervention, there was no statistically significant difference in the SCL‐90 scores between the two study groups (*P* > .05). After the intervention, the scores in both groups decreased for all subscales and indices of the SCL‐90. However, the results of this study show that, compared with conventional nursing methods, mindfulness decompression therapy had a somewhat more significant effect on the mental health status of nurses in the nucleic acid sampling area, with more positive SCL‐90 results observed. According to the SCL‐90 assessment, there were statistically significant differences in the scores for each factor between the control group and mindfulness group nurses (all *P* < .05). These are further detailed in Table 3.

**TABLE 3:** Comparison of SCL‐90 scores between the two groups of participants before and after intervention (*n* = 130).

Dimension		Control group (*n* = 65)	Mindfulness group (*n* = 65)	*t*	*P*
Somatization	Before	2.349 ± 0.534	2.168 ± 0.490	−1.811	.056
	After	1.987 ± 0.535	1.288 ± 0.179	15.181	<.001
	t*	−3.858	13.600		
	P*	<.001	<.001		
Obsessive–compulsive symptoms	Before	2.655 ± 0.482	2.463 ± 0.515	−1.354	.178
	After	2.348 ± 0.454	1.717 ± 0.243	14.03	<.001
	t*	−3.748	10.562		
	P*	<.001	<.001		
Interpersonal sensitivity	Before	2.323 ± 0.439	2.133 ± 0.496	−1.195	.234
	After	2.036 ± 0.431	1.362 ± 0.228	15.661	<.001
	t*	−3.766	11.378		
	P*	<.001	<.001		
Depression	Before	2.260 ± 0.421	2.124 ± 0.418	−1.063	.29
	After	2.050 ± 0.381	1.508 ± 0.144	13.626	<.001
	t*	−2.991	11.247		
	P*	.003	<.001		
Anxiety	Before	2.303 ± 0.472	2.174 ± 0.440	−1.787	.059
	After	2.018 ± 0.451	1.449 ± 0.172	13.69	<.001
	t*	−3.512	12.356		
	P*	.001	<.001		
Hostility	Before	2.333 ± 0.585	2.149 ± 0.583	−1.345	.071
	After	1.910 ± 0.577	1.444 ± 0.227	11.436	<.001
	t*	−4.153	9.092		
	P*	<.001	<.001		
Terror	Before	2.123 ± 0.449	1.954 ± 0.459	−1.886	.059
	After	1.791 ± 0.475	1.277 ± 0.278	12.926	<.001
	t*	−4.096	10.176		
	P*	<.001	<.001		
Paranoid	Before	2.072 ± 0.428	1.941 ± 0.421	−1.856	.066
	After	1.795 ± 0.475	1.428 ± 0.260	10.361	<.001
	t*	−3.493	8.351		
	P*	.001	<.001		
Psychosis	Before	2.029 ± 0.401	1.840 ± 0.396	−1.855	.066
	After	1.703 ± 0.445	1.222 ± 0.167	14.986	<.001
	t*	−4.391	11.609		
	P*	<.001	<.001		
Appetite and sleep disorders	Before	2.378 ± 0.546	2.264 ± 0.514	−0.936	.563
	After	2.202 ± 0.437	1.705 ± 0.401	8.005	<.001
	t*	−2.028	6.905		
	P*	.045	<.001		
SCL‐90 Overall average	Before	2.292 ± 0.372	2.130 ± 0.375	−1.966	.051
	After	1.997 ± 0.358	1.437 ± 0.038	18.422	<.001
	t*	−4.612	14.803		
	P*	<.001	<.001		

*Note*: t* and P* Results of intra‐group comparison before and after intervention. *t* and *P* are the results of the comparison between the two groups before and after the intervention.

### 3.3 Comparison of job burnout scores between the two groups of participants before and after the intervention

The results indicated that before the intervention, there were no statistically significant difference in the MBI‐GS scores of the two groups of nurses in each dimension (*P* > .05). After the intervention, the professional efficacy of the nurses in the control group was lower than before. Compared to the control group, the results revealed more significant positive effects in the mindfulness group in terms of emotional exhaustion, professional efficacy, and cynicism. According to the MBI‐GS assessment, the difference was statistically significant: emotional exhaustion (1.880 ± 0.637, 0.942 ± 0.575); professional effectiveness (1.036 ± 0.888, 4.044 ± 1.087); cynicism (2.992 ± 0.901, 0.469 ± 0.494), all *P* < .05. These data are further detailed in Table 4.

**TABLE 4:** Comparison of occupational burnout scores between the two groups of participants before and after intervention (*n* = 130).

Dimension		Control group (*n* = 65)	Mindfulness group (*n* = 65)	*t*	*P*
Emotional exhaustion	Before	16.145 ± 12.031	12.302 ± 11.484	1.863	.065
	After	1.880 ± 0.637	0.942 ± 0.575	8.814	<.001
	t*	9.546	7.966		
	P*	<.001	<.001		
Professional efficacy	Before	3.538 ± 1.178	3.579 ± 1.211	−1.500	.136
	After	1.036 ± 0.888	4.044 ± 1.087	14.350	<.001
	t*	14.269	26.639		
	P*	<.001	<.001		
Cynicism	Before After	3.424 ± 1.124 2.992 ± 0.901	3.655 ± 1.277 0.469 ± 0.494	−1.094 19.805	.276 <.001
	t*	2.419	18.755		
	P*	.017	<.001		
MBI‐overall average	Before After	3.965 ± 0.765 2.025 ± 0.066	3.770 ± 0.768 0.576 ± 0.256	1.451 44.119	.149 <.001
	t*	20.366	31.789		
	P*	<.001	<.001		

*Note*: t* and P* Results of intra‐group comparison before and after intervention. *t* and *P* are the results of the comparison between the two groups before and after the intervention.

## 4 DISCUSSION

We compared psychological states and job burnout of our two subject groups after 8 weeks of intervention. The results of mindfulness‐based stress reduction therapy were found to be better than those of routine intervention for nurses working in the nucleic acid sampling area. Our findings indicate that mindfulness decompression therapy is a practical approach to address burnout and declining mental health among nurses in nucleic acid sampling areas.

Previous studies have shown that the incidence of nurse burnout varies from 21.6% to 67.6%.^18^, ^19^, ^20^, ^21^ The populations in these studies included male nurses, new nurses, nurses of different specialties, nurses in different positions, and others. However, we must admit that the coronavirus pandemic has increased the burden on the global medical system, and healthcare providers are particularly vulnerable to emotional distress given their increased risk of exposure to the virus, concern about infecting and caring for their loved ones, and shortages of personal protective equipment. Longer work hours and involvement in emotionally and ethically‐fraught resource allocation decisions have also been found to contribute.^21^, ^22^, ^23^, ^24^ Airtight protective clothing throughout the entire process, a continuous long‐term, high‐risk working environment, and worries and fears about contracting COVID‐19 can easily lead to psychological problems for nucleic acid sampling nurses and cause job burnout. Therefore, it is essential to adopt effective psychological interventions to stabilize the mental states of clinical first‐line nucleic acid sampling nurses and maintain their psyches.

During the COVID‐19 epidemic, medical health workers had high prevalence rates of severe insomnia, anxiety, depression, somatization, Appetite and sleep disorders, and obsessive–compulsive symptoms. They also had risk factors for developing insomnia, anxiety, depression, obsessive–compulsive symptoms, and somatization.^25^, ^26^ The first finding of this study was that mindfulness decompression therapy has a positive effect on the mental health status of nurses. The total scores on the SCL‐90 and each dimension in our observation group were significantly lower than those in the control group. Mindfulness‐based stress reduction can therefore effectively relieve anxiety, depression, hostility, phobic anxiety, compulsion, and other psychological states of clinical front‐line nucleic acid sampling nurses. It can effectively enhance positive emotions and relieve negative ones, especially anxiety, depression, anger, and other negative emotions related to exposure to confirmed COVID‐19 patients.^27^

Our study also found that mindfulness decompression therapy measures positively affected nurses' job burnout in the clinical front‐line nucleic acid sampling area, which can be inferred from the burnout scores of the two groups after the intervention. Research shows that long‐term work with people as the objects of service is prone to job burnout, and frontline nurses are a high‐risk group for job burnout.^28^ Mindfulness decompression can effectively improve job burnout among first‐line nucleic acid‐sampling nurses. This may be because mindfulness decompression makes first‐line nucleic acid sampling nurses more responsible and mission‐orientated, and more dedicated so they can effectively overcome negative emotions.^29^ Many studies^27^, ^29^ have shown that mindfulness decompression can effectively alleviate the job burnout of nurses in various clinical positions, reduce emotional exhaustion, improve work attitudes, improve personal senses of accomplishment, and allow nurses to better devote themselves to work.^9^ Adequate support from managers and organizations can best support the resilience of front‐line nurses, including implementing flexible work environments, appropriate patient–nurse ratios, flexible work schedules, adequate supplies, and equipment (such as PPE), up‐to‐date information on the virus, and mental health resources. All of these can bring job security to nurses in the first line nucleic acid sampling area.

### 4.1 Limitations

This study has several limitations. First, the participants were limited to frontline nurses in the nucleic acid sampling area, which was a specific population and lacked broader representativeness. The study participants were also frontline nurses in a nucleic acid sampling area in a city in Hunan Province, China, making the results subject to geographical restrictions. Therefore, these results may not be representative of a broader population. A more comprehensive range of people should therefore be studied in the future. Second, the study compared a group from June 2020 to April 2021 and a group from May to December 2021. Over this period of time, nurses may have become more familiar with COVID‐19, and other changes may have taken place over time. Thus, there was also a selection bias in the two groups of subjects.

Third, this study was a retrospective analysis, and it was impossible to conclude causality. Mindfulness decompression therapy seems promising for reducing psychological distress among nurses. Future research should include randomized controlled trials with larger sample sizes and follow‐up studies. There should also be a focus on creative and effective ways to deliver mindfulness decompression therapy to nurses. Fourth, we did not measure previous mental health history because the participants were already working on the front lines when we started assessing their situation. We did not have pre‐COVID‐19 data to compare prior rates of stress, anxiety, and job burnout; thus, our interpretation of the data was only within the study timeframe. Finally, the study was conducted at a single point during China's second wave of the COVID‐19 outbreak (Delta). These findings may differ from those at other stages of China's pandemic and economic situation.

## 5 CONCLUSIONS

Mindfulness decompression therapy can effectively relieve the psychological pressure of nucleic acid sampling nurses and reduce the job burnout of nurses in this field. It is recommended that relevant management departments set up a psychological intervention team to provide regular mindfulness decompression counseling to nucleic acid sampling nurses to ensure the mental health of first‐line nucleic acid sampling nurses.

## AUTHOR CONTRIBUTIONS

Luo Lingxia: paper writing, routine nursing intervention, and mindfulness‐based stress reduction therapy; Zhang Jinghui: research design, thesis revision; Hou Jianmei, Peng Xiaobei, Xie Yanhui, Dong Huiqian: routine nursing intervention and mindfulness‐based stress reduction therapy, follow‐up, data collection; Ma Guiyuan, Peng Sha: data sorting, Statistical Analysis.

## FUNDING INFORMATION

This study was funded by the Changsha Municipal Natural Science Foundation in Hunan Province, China (grant no: kq2014298) and General Topics of the Hunan Administration on Traditional Chinese Medicine, China (grant no: D2022002).

## DISCLOSURE

*Approval of the research protocol*: This study was approved by the Ethics Committee of Xiangya Hospital Central South University, China (approval No. 202005402). *Informed Consent*: Written informed consent was obtained from all the participants. *Registry and registration*: N/A. of the study/trial N/A. *Animal studies*: N/A. *Conflict of interest*: The authors declare no conflict of interest associated with this manuscript.

## Supporting information


Video S1.

Video S2.

## Data Availability

The data supporting the findings of this study are available from the corresponding author upon reasonable request.
